# Epidemiological Survey of Porcine Circovirus Types 2 and 3 in Liaoning Region of China and Preparation of Monoclonal Antibodies Against PCV3 Cap Protein

**DOI:** 10.3390/vetsci13030218

**Published:** 2026-02-25

**Authors:** Jiahui Liu, Siyao Min, Delong Li, Shiqi Zhang, Jiahuan Zhong, Wenqian Wang, Xinyang Song, Xiaoxi Sun, Changde Wu, Xinghe Wang

**Affiliations:** College of Animal Science and Veterinary Medicine, Shenyang Agricultural University, Shenyang 110866, China; 2025200250@stu.syau.edu.cn (J.L.); 13304996270@163.com (S.M.); 2023220609@stu.syau.edu.cn (D.L.); 2023240835@stu.syau.edu.cn (S.Z.); 2023240844@stu.syau.edu.cn (J.Z.); 2024220631@stu.syau.edu.cn (W.W.); 2024220630@stu.syau.edu.cn (X.S.); 18222459410@163.com (X.S.)

**Keywords:** porcine circovirus, PCV3 *Cap* gene, genetic evolution analysis, indirect ELISA method, monoclonal antibody

## Abstract

*Porcine circovirus* (PCV) is a major viral pathogen associated with multiple systemic diseases in pigs, inflicting significant economic losses to the global swine industry. To investigate the epidemiology and genetic evolution of porcine circovirus in Liaoning Province, China, PCV was detected by qPCR in 1224 clinical samples. Subsequent genetic evolution analysis of the *Cap* gene was conducted, and an indirect ELISA and monoclonal antibody were established. The results demonstrated PCV2 and PCV3 positivity rates of 14.13% and 21.90%, respectively, with a coinfection rate of 4.08%. All six sequences were identified as belonging to the PCV3b subtype. A representative PCV3 strain was expressed in a prokaryotic expression system and used to immunize 6-week-old female BALB/c mice, resulting in serum antibody titers reaching 1:512,000. The positive hybridoma cell line 3E6 was selected and identified as expressing IgM heavy chains and κ light chains. The prepared monoclonal antibody 3E6 exhibited specific reactivity with the Cap protein. Collectively, this study elucidates the recent epidemiological status and evolutionary characteristics of PCV in pig populations in Liaoning Province, thereby providing an important theoretical basis and reference data for disease prevention, control, diagnosis, and vaccine development.

## Simple Summary

*Porcine circovirus* (PCV) is a major viral pathogen associated with multiple systemic disorders in pigs, inflicting substantial economic losses on the global swine industry. In 2024, 1224 clinical samples were collected from 21 large-scale pig farms in Liaoning Province and detected for PCV2 and PCV3 using quantitative polymerase chain reaction (qPCR). The results demonstrated PCV2 and PCV3 positivity rates of 14.13% and 21.90%, respectively, with a coinfection rate of 4.08%. Six *Cap* gene sequences were obtained by PCR amplification and sequencing, and their sequences were classified as belonging to the PCV3b subtype. Furthermore, the PCV3 Cap protein was heterologously expressed in a prokaryotic system, an indirect ELISA was established, and a specific monoclonal antibody (3E6) targeting this protein was successfully developed. Collectively, this study elucidates the epidemiological characteristics and genetic evolutionary patterns of PCV in Liaoning Province, providing crucial theoretical support and experimental evidence for the prevention and control of PCV3, as well as for the development of diagnostic methods and vaccines against this virus.

## 1. Introduction

*Porcine circovirus* (PCV) is a small, non-enveloped virus that belongs to the genus *Circovirus* within the family *Circoviridae*. With a particle diameter of approximately 14–17 nm and a regular icosahedral structure, it is currently considered the smallest known single-stranded circular DNA virus infecting mammals [1]. Currently, porcine circoviruses are divided into four distinct species, namely *Porcine circovirus* 1 (PCV1), *Porcine circovirus 2* (PCV2), *Porcine circovirus 3* (PCV3), and *Porcine circovirus 4* (PCV4) [2]. PCV1 was first isolated from the PK-15 porcine kidney cell line in 1974, and studies confirmed its non-pathogenicity [1]. PCV2 was discovered in Canada in 1995 and clinically causes a series of porcine circovirus-associated diseases (PCVAD), including postweaning multisystemic failure syndrome [3]. PCV3 was first identified in the United States in 2016 and subsequently spread rapidly worldwide. PCV3 has been widely reported in multiple countries, including China, Japan, South Korea, Russia, Denmark, Italy, and Spain [4,5,6,7,8]. Multiple studies indicate that PCV3 can cause reproductive disorders in sows, postweaning multisystemic wasting syndrome (PMWS), porcine dermatitis and nephropathy syndrome (PDNS), and multisystemic inflammation. It has now become one of the most damaging swine diseases threatening the livestock industry [9,10]. In 2019, a novel porcine circovirus was first identified in diseased pigs in Hunan, China, and designated PCV4. This virus causes respiratory and reproductive diseases in pigs [2,11].

The PCV3 genome spans approximately 2000 bp and contains three open reading frames (ORFs). ORF1, which is 896 bp in length, encodes a 296-amino-acid replication protein (Rep). ORF3 is 693 bp long and encodes a 230-amino-acid protein, the specific function of which remains unclear [9]. ORF2 spans 645 bp and encodes the Cap protein, comprising 214 amino acids. The Cap protein serves as the sole structural protein of PCV3 [12,13] and is also the primary immunogenic protein, harboring major antigenic determinants that induce the production of virus-specific neutralizing antibodies [14,15]. Based on two mutation sites in the Cap protein (A24V and R27K), PCV3 can be classified into three subtypes: PCV3a, PCV3b, and PCV3c [16]. Compared with the PCV2 *Cap* gene, the nucleotide and amino acid sequence similarities are only 36% and 26%, respectively [12,13]. Currently, PCV2 prevention and control primarily rely on inactivated or subunit vaccines targeting the PCV2a genotype. Commercial vaccines such as Ingelvac CircoFLEX^®^ and Porcilis^®^ PCV have been widely used [17]. Existing studies have demonstrated that PCV2 vaccines do not confer cross-protection against PCV3, and no commercial vaccines targeting PCV3 are currently available [18,19]. Therefore, epidemiological investigations and phylogenetic analyses based on the PCV3 *Cap* gene are of great importance for the prevention, control, and development of PCV3 vaccines.

Both PCV2 and PCV3 primarily replicate in lymphoid tissues, leading to destruction of lymphoid follicles, immunosuppression, and increased susceptibility of pigs to secondary infections, which further exacerbate clinical symptoms and complicate disease control efforts [9,20]. Previous studies in China have mainly focused on overall sequence homology and genotype classification of the PCV3 Cap gene, with limited attention paid to mutations in key functional domains, and studies focusing on PCV3 infection and its immunological characteristics in Northeast China remain more limited [21,22,23]. In this study, real-time quantitative PCR technology was employed to detect PCV2 and PCV3 infections in 1224 clinical samples collected from 21 large-scale pig farms in Liaoning Province in 2024. Additionally, genetic evolutionary analysis of the PCV3 Cap gene was conducted. Additionally, an indirect ELISA and monoclonal antibodies were developed for the PCV3 Cap protein. Further investigations into the biological functions and immunogenicity of recombinant PCV3 Cap proteins provided foundational data for PCV3 detection and vaccine development.

## 2. Materials and Methods

### 2.1. Clinical Samples, Cells, Positive Serum Experimental Animals, and Primary Reagents

In 2024, a total of 1224 clinical samples—including lungs, spleen, kidneys, inguinal lymph nodes, nasal swabs, and serum—were collected from 21 large-scale farms in Liaoning Province. Samples were collected from three types of pigs (finishing pigs, sows, and piglets) showing clinical signs, such as diarrhea, rapid breathing, and reproductive disorders that are suspected to be infected. These samples were collected in collaboration with Liaoning Pet Companion Biotechnology Co., Ltd. (Shenyang, China) and stored in the Pathology Laboratory of the College of Animal Science and Veterinary Medicine at Shenyang Agricultural University.

The 293T and Sp2/0 cell lines were stored in our laboratory. Both cell lines were cultured at 37 °C in a 5% CO_2_ incubator (Thermo, Waltham, MA, USA). The recombinant plasmid containing the *Cap* gene was obtained using the ClonExpress Ultra One Step Cloning Kit (C115, Vazyme, Nanjing, China). PCV2-, PRRSV-, and PEDV-positive sera are all stored in our laboratory. PCV2-, PRRSV-, PEDV-positive, and other pathogen-positive sera are all stored in our laboratory. The health experimental animals were 6-week-old female SPF-grade BALB/c mice purchased from Changsheng Biotechnology Co., Ltd., Benxi, Liaoning, China (SCXK(Liao)2020-0001). The body weight of the mice used in the experiment was 20 ± 2 g. The animal study protocol was approved by the Animal Welfare and Ethics Committee of Shenyang Agricultural University (Approval No: SNLL2504501).

### 2.2. Primer Design and Synthesis

Based on the PCV3 genome sequence registered in PCV3/CN/Fujian-12/2016 (KY075987), primers were designed for PCV3 detection (PCV3-F1/PCV3-R1) and for amplification of the full-length PCV3 *Cap* gene (PCV3-F2/PCV3-R2) [24]. Using *Cap* gene sequences from Liaoning Province as templates, we constructed a truncated recombinant expression plasmid. All primers were synthesized by the company (Table 1) (Sangon, Shanghai, China).

### 2.3. qPCR Detection and Positive Rate Statistics for PCV

Viral genomic DNA was extracted using the Viral DNA/RNA Mini Kit (Vazyme, Nanjing, China) according to the manufacturer’s instructions. Real-time fluorescent detection of PCV2 and PCV3 was performed using the Real-Time PCR PCV2/PCV3 Detection Kit (IDEXX, Westbrook, ME, USA). The statistical software used for the positivity rate analysis is Microsoft Excel (Microsoft Corp., Redmond, WA, USA). PCV3 qPCR-positive samples were randomly selected for subsequent *Cap* gene amplification and cloning.

### 2.4. Amplification and Cloning of the PCV3 Cap Gene

PCV3 detection was conducted using primers PCV3-F1/PCV3-R1. The PCR cycling conditions were as follows: initial denaturation at 95 °C for 4 min; 34 cycles of denaturation at 95 °C for 30 s, annealing at 55 °C for 30 s, and extension at 72 °C for 30 s; followed by a final extension at 72 °C for 5 min. PCR products were analyzed by electrophoresis on a 1% agarose gel. *Cap* gene amplification was performed using primers PCV3-F2/PCV3-R2 under identical reaction conditions, except that the annealing temperature was adjusted to 63 °C. Purified PCR products were ligated into the pUCm-T vector and subsequently transformed into *Escherichia coli* DH5α competent cells. Select 3–4 single colonies and culture them at 37 °C for 12 h in LB liquid medium with ampicillin. After extraction, the recombinant plasmid was digested with BamHI and EcoRI to confirm its identity.

### 2.5. PCV3 Cap Gene Sequence Analysis

Confirmed recombinant plasmids were submitted for sequencing (Sangon, Shanghai, China). Forty-four representative PCV3 *Cap* gene sequences were retrieved from NCBI. Nucleotide and amino acid sequence homology analyses were performed using MegAlign 11.1.0 (DNASTAR Inc., Madison, WI, USA). Phylogenetic analysis was conducted using MEGA 11 software to determine the genetic subtypes of circulating strains. Evolutionary analyses were conducted with 1000 bootstrap replicates to identify clustering and evolutionary relationships. In addition, MegAlign, CLUSTALW, ESPript 3.0, and the WebLogo 3.7.9 online tool were employed to compare Cap protein amino acid sequences with those of three classical reference strains, thereby elucidating the molecular characteristics and genetic variation of circulating PCV3 strains in Liaoning Province.

### 2.6. Construction of a Recombinant Expression Plasmid for the Truncated Cap Gene in Prokaryotes

A recombinant expression plasmid encoding a truncated *Cap* gene enriched in N-terminal arginine residues (aa 1–31) was constructed. Signal peptide and transmembrane domains of the truncated Cap protein were predicted using the online tools SignalP-4.1 and TMHMM-2.0. The PCR amplification conditions for the truncated *Cap* gene were as follows: pre-denaturation at 95 °C for 4 min; 35 cycles of denaturation at 95 °C for 30 s, annealing at 60 °C for 30 s, and extension at 72 °C for 30 s; followed by a final extension at 72 °C for 5 min. The amplified products were purified and ligated into the BamHI and XhoI-linearized pET-32a(+) vector. Recombinant plasmids were extracted, identified by double-restriction enzyme digestion, and validated by sequencing (Sangon, Shanghai, China). The truncated PCV3 *Cap* gene was cloned and inserted at the downstream of the lac operator in the prokaryotic expression pET-32a (+) vector, and the recombinant protein expression was induced by isopropyl β-D-1-thiogalactopyranoside (IPTG). In addition, the full-length *Cap* gene (645 bp) eukaryotic expression plasmid pcDNA3.1-PCV3 Cap was synthesized by a commercial provider (Sangon, Shanghai, China).

### 2.7. Induced Expression and Condition Optimization of Recombinant Plasmids

The recombinant plasmid pET-32a-PCV3 Cap∆N was transformed into *E. coli* BL21 (DE3) competent cells. Positive clones were selected and cultured in LB liquid medium supplemented with ampicillin at 37 °C for 12 h. Cultures were diluted 1:100 and incubated at 37 °C with shaking at 200 rpm until the optical density at 600 nm reached 0.6. IPTG was added at the logarithmic growth phase to a final concentration of 0.5 mM. Induction was carried out at 37 °C for 6 h, 25 °C for 12 h, and 16 °C for 16 h, respectively. IPTG-free controls and empty vector pET-32a (+) controls were included to evaluate expression patterns and determine optimal induction conditions. Under optimal induction conditions, IPTG was added at final concentrations of 0, 0.2, 0.5, 0.8, 1.0, 1.2, and 1.5 mM to determine the optimal inducer concentration.

### 2.8. Purification and Renovation of Recombinant Proteins

Bacteria were harvested by centrifugation at 4 °C and resuspended in bacterial protein lysis buffer containing PBS (B540149, Sangon, Shanghai, China). Ultrasonication was performed at 500 W with cycles of 4 s on and 8 s off for a total duration of 10 min by a cell ultrasonic homogenizer (XM-650T, Ultrasonics, Jiangsu, China). Following centrifugation, the supernatant was defined as the soluble protein fraction. The pellet was resuspended in inclusion body lysis buffer containing urea (B540148, Sangon, Shanghai, China) and subjected to ultrasonication and centrifugation under identical conditions, and the resulting supernatant was defined as the inclusion body protein fraction. Protein samples were mixed with 5× loading buffer and heated at 100 °C for 10 min. Protein expression was analyzed by 10% SDS-PAGE and Western blot.

### 2.9. SDS-PAGE and Western Blot

The purified and refolded PCV3 Cap protein was subjected to 10% SDS-PAGE and subsequently transferred to a PVDF membrane. After blocking with a protein-free rapid blocking solution (PS108P; Epizyme, Shanghai, China) for 20 min, the membrane was washed once with TBST. Anti-His monoclonal antibody was used as the primary antibody at a dilution of 1:5000 and incubated at 4 °C for 12 h. The membrane was washed three times with TBST, followed by incubation with goat anti-mouse IgG-HRP (1:10,000) as the secondary antibody at 37 °C for 1 h. After three additional washes with TBST, ECL chemiluminescent substrate was applied for signal detection using the Omni-ECL™ Basic Chemiluminescence Detection Kit (SQ202; Epizyme, Shanghai, China). Protein bands were visualized using a multifunctional imaging system (Analytik Jena AG, Thuringia, Germany).

### 2.10. Optimization of Optimal Reaction Conditions for Indirect ELISA

Following standard indirect ELISA procedures, the optimal coating conditions for the PCV3 Cap protein were determined using a matrix titration method. Each well was coated with 100 μL of recombinant protein at different concentrations and incubated at 4 °C overnight. Then wash the plate three times with PBST, followed by blocking with ELISA Plate Blocking Buffer (E-ELIR-003; Elabscience, Wuhan, China) at 37 °C. After washing, the plates were incubated with positive serum at serial dilutions as the primary antibody at 37 °C. Diluted goat anti-mouse HRP-IgG was then added as the secondary antibody and incubated at 37 °C. After washing, one-component TMB substrate solution (E-IR-R201; Elabscience, Wuhan, China) was added and incubated at 37 °C in the dark, and the reaction was terminated by the addition of 50 μL stop solution (E-ELIR-006; Elabscience, Wuhan, China). OD_450_ values were measured using a microplate reader (Infinite F200; Tecan, Männedorf, Switzerland). P/N value ≥ 2.1 was considered positive, where P represents the OD_450_ value of the positive serum, and N represents the OD_450_ value of the negative control. The optimal working concentration was defined as the concentration that yielded the maximum P/N ratio.

### 2.11. Determination of the Cut-Off Value for the Indirect ELISA Method

The ELISA cut-off value was determined using 60 pig serum samples identified as negative by qPCR. Based on statistical principles, results were classified as positive when S/P ≥ X + 3SD, negative when S/P < X + 2SD, and suspect when values fell between these thresholds. Repeatability tests were conducted on the established ELISA method to determine the coefficient of variation.

### 2.12. Specificity Testing and Repeatability Testing of the Indirect ELISA Method

Positive serum samples for PRRSV, CSFV, PRV, PEDV, PCV2, and other pathogens stored in the laboratory were tested to validate the specificity of this detection method. The sample-to-positive (S/P) ratio was calculated using the following formula: S/P = (OD_450_ sample − OD_450_ negative control)/(OD_450_ positive control − OD_450_ negative control).

PCV3 Cap proteins prepared from the same and different batches were used to coat 96-well plates as antigens. Three PCV3-positive and PCV3-negative serum samples were randomly selected for three replicate tests. The coefficient of variation for intra-batch and inter-batch replicate tests was calculated based on OD_450_ values. OD_450_ values were measured using a microplate reader (Infinite F200; Tecan, Switzerland)

### 2.13. Clinical Testing of the Indirect ELISA Method

To verify the clinical applicability, sensitivity, and specificity of the ELISA method, we detected 150 clinical serum samples (A random sample of 21 large-scale pig farms in Liaoning Province) by both ELISA and qPCR.

### 2.14. Mouse Immunization

After concentration, the PCV3 Cap recombinant protein was emulsified with an equal volume of adjuvant, consisting of Freund’s complete adjuvant for the primary immunization and Freund’s incomplete adjuvant for the second and third immunizations. The experiment was divided into two groups: immunized (n = 6) and negative control (n = 6). The antigen–adjuvant mixture was administered subcutaneously at multiple sites to 6-week-old female BALB/c mice at a dose of 100 μg per mouse per immunization. A total of four immunizations were performed: the first three immunizations were administered at 14-day intervals. Seven days after the third immunization, a booster dose of 50 μg of recombinant protein per mouse was administered via intraperitoneal injection. Blood samples were collected from the tail vein one day prior to each immunization, and serum was separated and stored at −80 °C in an ultra-low-temperature freezer (DW-86W208; Sigma, St. Louis, MO, USA).

### 2.15. Mouse Serum Antibody Titer Assay

An indirect enzyme-linked immunosorbent assay (ELISA) was performed to determine the titers of antibodies against the PCV3 Cap protein in mouse sera. The PBST-diluted Cap recombinant protein and serum from immunized mice were used as antigens and primary antibodies, respectively, for the determination of mouse serum antibody titers. OD_450_ values were measured using a microplate reader (Infinite F200; Tecan, Switzerland). All reaction conditions were detected according to the optimized ELISA conditions.

### 2.16. Preparation of Monoclonal Antibodies

Ninety-six–well plates were pre-seeded with peritoneal macrophages using HAT selection medium (HAT Medium Supplement (H0262), Sigma, USA; RPMI-1640, 20% FBS, 1% antibiotic-antimycotic, Gibco, Waltham, MA, USA). Three days after the booster immunization, mice were euthanized, and splenocytes were harvested and fused with SP2/0 myeloma cells. The fused cells were incubated at 37 °C in a humidified incubator. On day 10 post-fusion, cultures were switched to HT medium for continued selection. (HT Medium Supplement (H0137) Sigma, USA; RPMI-1640, 20% FBS, 1% antibiotic-antimycotic, Gibco, USA). When microscopic observation showed that cell clusters occupied more than one-quarter of the visual field in individual wells, hybridoma screening was performed using the indirect ELISA described above. Positive hybridoma clones were subcloned three successive times, expanded, and continuously passaged. Antibody isotypes were determined using a mouse monoclonal antibody subclass identification kit, and ascites fluid was generated in mice to obtain high-titer monoclonal antibodies.

### 2.17. Immunofluorescence Assay

Actively growing HEK 293T cells were seeded into 6-well plates (DMEM, 10% FBS, 1% antibiotic-antimycotic, Gibco, USA). At approximately 70% confluence, cells were transfected with the eukaryotic recombinant plasmid pcDNA3.1-PCV3 Cap or the pcDNA3.1 empty vector. After 48 h of incubation, cells were washed three times with PBS, fixed with 4% paraformaldehyde for 10 min, and permeabilized. Cells were then incubated overnight at 4 °C with either 3E6 monoclonal antibody-containing hybridoma supernatant or diluted mouse positive serum (1:400) as the primary antibody. Following three PBS washes, FITC-conjugated goat anti-mouse IgG (1:200) was applied as the secondary antibody and incubated at 37 °C for 1 h. Finally, cells were counterstained with 4′,6-diamidino-2-phenylindole (DAPI) and examined under an inverted fluorescence microscope (Leica, Wetzlar, Germany).

## 3. Results

### 3.1. PCV2 and PCV3 Positive Rates in Liaoning Province

Among the 1224 clinical samples collected, the PCV3-positive rate was 21.90% (268/1224), the PCV2-positive rate was 14.13% (173/1224), and the PCV2/PCV3 coinfection rate was 4.08% (50/1224). The PCV3 positivity rate peaked in July and December, while PCV2 positivity was highest in January and February. Coinfection with both viruses reached its highest level in January (Table 2). The prevalence of PCV3, PCV2, and coinfection was higher in the Shenyang and Dalian regions of Liaoning Province (Table 3). In addition, relatively high PCV detection rates were observed in serum, Lymph node, and nasal swab samples (Table 4).

### 3.2. PCV3 Cap Gene Amplification and Identification

**Figure 1:**
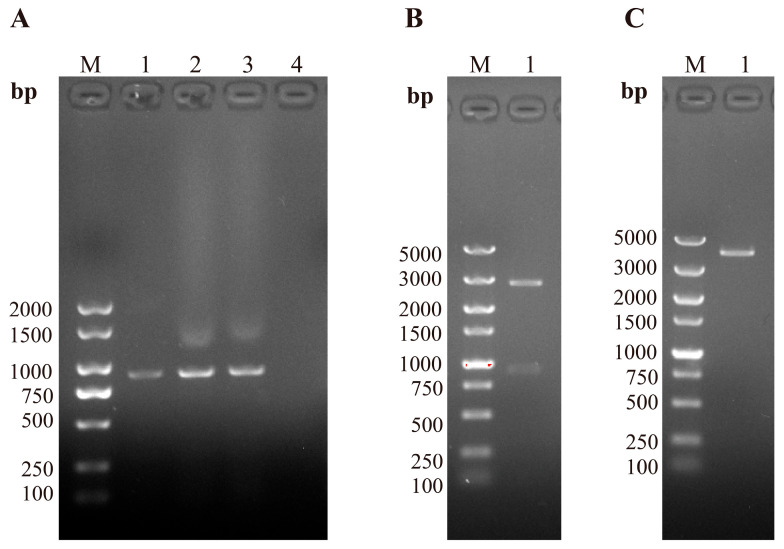
PCR amplification and restriction enzyme digestion analysis of the PCV3 *Cap* gene. (**A**) PCR amplification of the full-length PCV3 *Cap* gene from qPCR-positive clinical samples. M: DNA marker; lanes 1–3: PCV3-positive samples; lane 4: negative control. (**B**) Double restriction enzyme digestion of the recombinant plasmid pUCm-T-PCV3-Cap. M: DNA marker; lane 1: digested pUCm-T-PCV3-Cap plasmid. (**C**) Single restriction enzyme digestion of the recombinant plasmid pUCm-T-PCV3-Cap. M: DNA marker; lane 1: digested pUCm-T-PCV3-Cap plasmid.

DNA extracted from qPCR-positive samples was used as the template for PCR amplification, and the resulting products were analyzed by 1% agarose gel electrophoresis. A specific amplification band was observed at approximately 870 bp (Figure 1A). Recombinant plasmids containing the target fragment were extracted and verified by restriction enzyme digestion. Following double enzyme digestion, two expected fragments of 2773 bp and 870 bp were observed (Figure 1B). Single enzyme digestion produced a linear fragment of approximately 3700 bp (Figure 1C), confirming the successful insertion of the PCV3 *Cap* gene into the pUCm-T vector.

### 3.3. Genetic Evolution Analysis of the PCV3 Cap Gene

**Figure 2:**
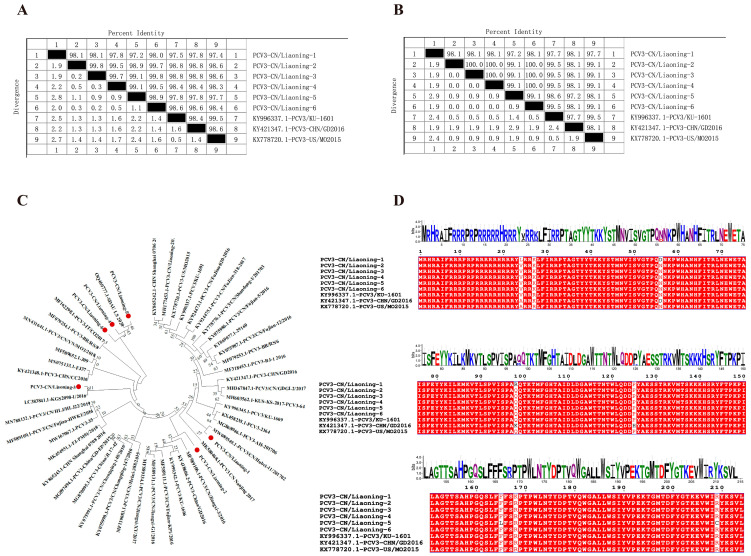
Genetic Evolution Analysis of the PCV3 *Cap* Gene in Liaoning Province. (**A**) Nucleotide sequence identity analysis of the PCV3 *Cap* gene. Nucleotide sequence identities between six PCV3 sequences and representative reference strains from the United States, South Korea, and China are shown as percentage identity (%). (**B**) Amino acid sequence identity analysis of the PCV3 Cap protein. Amino acid sequence identities between the six sequences and reference strains are expressed as percentage identity (%). (**C**) Phylogenetic analysis based on the PCV3 *Cap* gene. Sequence alignment and phylogenetic tree construction were performed using the Neighbor-Joining method in MEGA software, with bootstrap values based on 1000 replicates. Filled circles (•) indicate PCV3 strains identified in this study; reference strains are labeled with their GenBank accession numbers. (**D**) Multiple sequence alignment and conservation analysis of the PCV3 Cap protein. Amino acid alignment was conducted using MegAlign software. Variable residues are highlighted, with mutated sites indicated in red and conserved residues shown in white. Conservation scores (bits) reflect amino acid conservation, and residue numbers are indicated above the alignment.

Six *Cap* gene sequences of PCV3 were obtained by sequencing, each 645 bp in length, and were designated PCV3-CN/Liaoning-1 to PCV3-CN/Liaoning-6. Nucleotide sequence identity among the *Cap* genes of the six sequences ranged from 97.2% to 99.8%. Comparison with three representative domestic and international PCV3 reference strains showed nucleotide identities ranging from 97.4% to 98.8%. Among the sequences, PCV3-CN/Liaoning-1 exhibited the lowest nucleotide identity (97.4%) with PCV3-US/MO2015, the earliest PCV3 strain identified in the United States. In contrast, PCV3-CN/Liaoning-2 and PCV3-CN/Liaoning-3 showed the highest nucleotide identity (98.8%) with PCV3-CHN/DG2016 and PCV3/KU-1601, which were first isolated in China and South Korea in 2016 (Figure 2A). Amino acid sequence identity analysis demonstrated that the six sequences shared identities ranging from 97.2% to 99.5% with the three reference PCV3 Cap proteins. The lowest amino acid identity (97.2%) was observed between PCV3-CN/Liaoning-5 and PCV3-CHN/DG2016. PCV3-CN/Liaoning-2, PCV3-CN/Liaoning-3, PCV3-CN/Liaoning-4, and PCV3-CN/Liaoning-6 exhibited 100% amino acid identity and showed the highest overall similarity (99.5%) to PCV3/KU-1601 (Figure 2B).

Phylogenetic analysis based on the *Cap* gene indicated that all six PCV3 sequences belonged to the PCV3b genotype, whereas PCV3a and PCV3c genotypes were not detected. These findings suggest that PCV3b is currently the predominant circulating genotype in Liaoning Province, China. Further analysis showed that PCV3-CN/Liaoning-5 was most closely related to the SDAU_LS_2020 strain, while PCV3-CN/Liaoning-3 clustered most closely with the reference strain KGS2098-1/2016, with both belonging to the same clade. Although PCV3-CN/Liaoning-2 clustered within the PCV3b clade, it exhibited the greatest phylogenetic divergence among the six sequences (Figure 2C), suggesting ongoing genetic diversification of the PCV3 *Cap* gene in this region.

The amino acid sequence of the Cap protein deduced from the *Cap* gene consists of 215 amino acids. Compared with the three reference strains, the Cap protein of the six sequences carried amino acid mutations at positions 24, 27, 56, 98, 125, 165, 168, and 209. Among the four sequences from PCV3-CN/Liaoning-2 to -6, six identical mutation sites were identified: A24V, R27K, D56N, K98Q, H125P, and R168K. PCV3-CN/Liaoning-1 harbored four specific mutations (A24L, N56D, Q98R, and R168K), whereas PCV3-CN/Liaoning-5 carried two unique mutations (F165L and R209C). Further alignment showed that all six isolates shared two common mutation sites, T77S and Y104F. Of these mutations, four (A24V, R27K, T77S, and Y104F) were located in hypervariable regions (Figure 2D). A conservation analysis using WebLogo 3.7.9 revealed marked variability across regions of the Cap protein, while several key residues remained highly conserved (Figure 2D). B-cell linear epitope prediction using the BepiPred-2.0 tool identified potential epitope regions at amino acid positions 5–28, 43–62, 72–83, 96–107, 115–162, 171–180, and 191–211. Seven mutation sites identified in the sequences were located within predicted B-cell epitope regions, which may affect antigenicity and contribute to immune evasion.

### 3.4. Construction of the Recombinant Plasmid pET-32a-PCV3 Cap∆N

**Figure 3:**
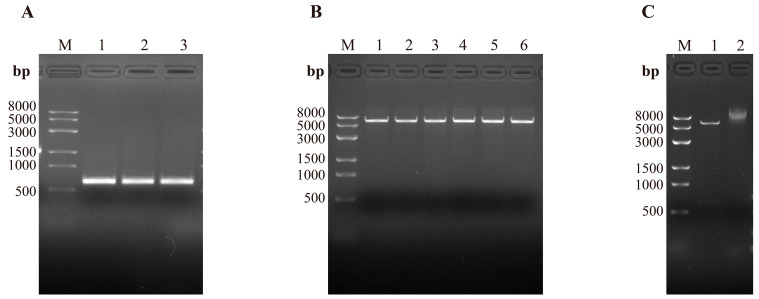
Construction and verification of the recombinant pET-32a-PCV3 Cap∆N plasmid. (**A**) PCR amplification of the truncated PCV3 *Cap* gene. M: DNA marker (8000 bp); lanes 1–3: positive samples. (**B**) Restriction enzyme digestion of the pET-32a vector for linearization. M: DNA marker; lanes 1–6: linearized pET-32a vector. (**C**) Double restriction enzyme digestion analysis of the recombinant pET-32a-PCV3 Cap∆N plasmid. M: DNA marker; lane 1: Recombinant plasmid pET-32a-PCV3 Cap∆N after double digestion; lane 2: recombinant pET-32a-PCV3 Cap∆N plasmid. Correct digestion patterns and sequencing analysis confirmed successful plasmid construction. The restriction enzymes used for vector linearization and double digestion are both BamHI and XhoI.

Four PCV3 strains exhibiting 100% amino acid sequence identity were identified in this study and were considered representative of the predominant circulating PCV3 Cap amino acid sequences in Liaoning Province. Accordingly, the amino acid sequence of PCV3-CN/Liaoning-2 was selected as the template for subsequent recombinant protein expression. Bioinformatic analysis demonstrated that the truncated *Cap* gene fragment was 549 bp in length (Figure 3A). The linearized length of the pET-32a vector is approximately 5900 bp (Figure 3B). Double restriction enzyme digestion produced the expected fragment pattern (Figure 3C), and sequencing analysis confirmed the successful construction of the recombinant pET-32a-PCV3 Cap∆N expression plasmid.

### 3.5. Induction and Purification of Recombinant Protein

**Figure 4:**
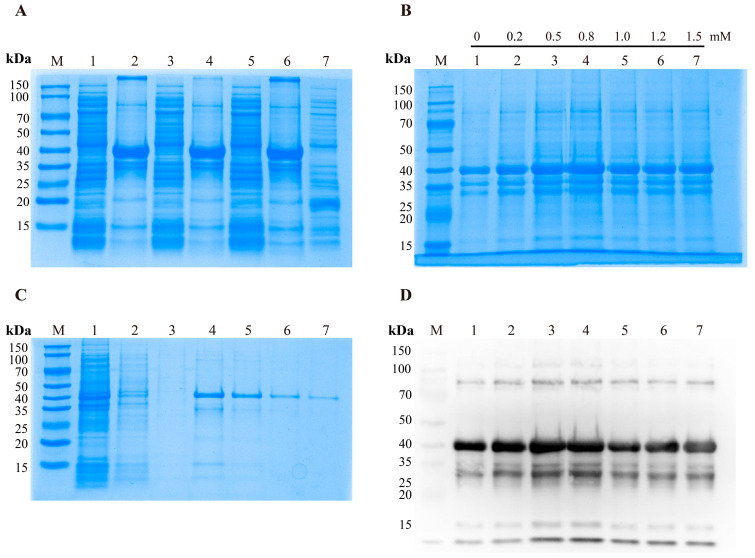
Expression, purification, and immunoblot analysis of the recombinant PCV3 Cap protein. (**A**) SDS–PAGE analysis of recombinant PCV3 Cap protein expressed in *E. coli* BL21 (DE3) at different induction temperatures. M: protein molecular weight marker (15–150 kDa); lane 1: supernatant induced at 16 °C; lane 2: pellet induced at 16 °C; lane 3: supernatant induced at 25 °C; lane 4: pellet induced at 25 °C; lane 5: supernatant induced at 37 °C; lane 6: pellet induced at 37 °C; lane 7: vector pET-32a Precipitate. (**B**) SDS–PAGE analysis of recombinant PCV3 Cap protein induced with different IPTG concentrations at the optimal induction temperature. M: protein molecular weight marker (15–150 kDa); lanes 1–7: induction with 0,0.2, 0.5, 0.8, 1.0, 1.2, and 1.5 mM IPTG, respectively. (**C**) SDS–PAGE analysis of purified recombinant PCV3 Cap protein. M: protein molecular weight marker (15–150 kDa); lane 1: flow-through fraction; lanes 2–3: wash fractions; lanes 4–7: elution fractions. (**D**) Western blot analysis of recombinant PCV3 Cap protein induced with different IPTG concentrations at the optimal temperature. M: protein molecular weight marker (15–150 kDa); lanes 1–7: induction with 0, 0.2, 0.5, 0.8, 1.0, 1.2, and 1.5 mM IPTG, respectively.

The recombinant plasmid was transformed into *E. coli* BL21 (DE3) competent cells for protein expression. A protein band of approximately 40 kDa was detected under induction conditions, whereas no corresponding band was observed in the pET-32a empty vector. The recombinant Cap protein was predominantly expressed as inclusion bodies (Figure 4A). Optimal induction conditions were determined to be incubation at 16 °C with 0.8 mM IPTG for 16 h (Figure 4A,B). Western blot analysis confirmed the identity of the recombinant protein and demonstrated specific reactivity with anti-His tag monoclonal antibodies (Figure 4B,D). Under optimized expression conditions, the recombinant protein was induced, harvested, and sonicated, after which the inclusion bodies were collected. The Cap protein was subsequently purified by nickel affinity chromatography and refolded. SDS-PAGE analysis revealed a single protein band at approximately 40 kDa, indicating high purity of the recombinant protein (Figure 4C).

### 3.6. Establishment of an Indirect ELISA Method

**Figure 5:**
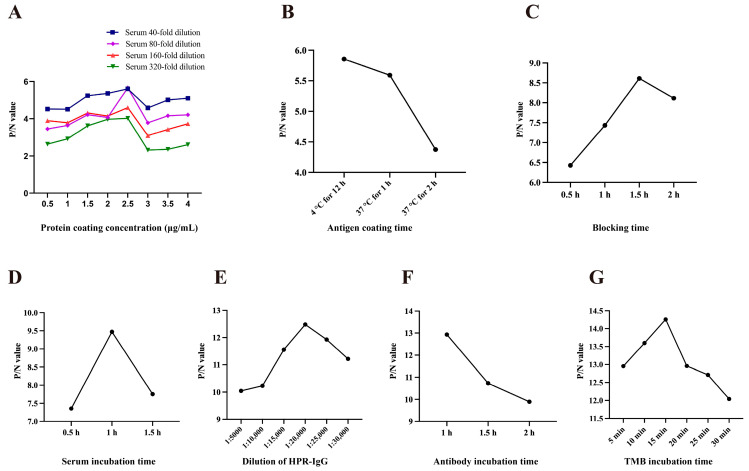
Optimized Conditions for Indirect ELISA. (**A**) Optimization of protein coating concentration versus serum dilution. (**B**) Optimization of antigen coating time. (**C**) Optimization of blocking time. (**D**) Optimization of positive serum reaction time. (**E**) Optimization of HRP-conjugated goat anti-mouse IgG dilution. (**F**) Optimization of HRP-conjugated goat anti-mouse IgG reaction time. (**G**) Optimization of TMB chromogen reaction time.

The optimized ELISA conditions are as follows: antigen coating concentration of 2.5 μg/mL; optimal serum dilution of 1:80; antigen coating time of 12 h at 4 °C; blocking time of 1.5 h; serum incubation time of 1 h; HRP-conjugated goat anti-mouse IgG dilution at 1:20,000 with an incubation time of 1 h; and substrate incubation time of 15 min (Figure 5A–G). A sample is considered positive when OD_450_ ≥ 0.412, negative when OD_450_ < 0.36, and suspicious when the value falls between these two thresholds. The specificity of this method was verified using positive sera from different viruses preserved in laboratories. The OD_450_ value for negative serum was 0.163, while the OD_450_ value for positive serum was 1.021. The results demonstrated that this ELISA kit reacts exclusively with PCV3-positive sera and does not produce antigen–antibody reactions with any other viruses (Table 5). The coefficient of variation within batches ranged from 2.03% to 4.94% (Table 6), while the coefficient of variation between batches ranged from 3.63% to 8.89% (Table 7). Both values were below 10%, indicating that this testing method exhibits good repeatability. Clinical testing shows an overall agreement rate of 94.0% (141/150) between the ELISA method and qPCR. Specifically, the agreement rate for positive samples was 91.9% (57/62), while that for negative samples was 95.5% (84/88). The optimal ELISA method was evaluated in three independent replicate experiments, and the results were analyzed based on mean values to ensure reliability and reproducibility.

### 3.7. Monoclonal Antibody Preparation and Subtype Identification

**Figure 6:**
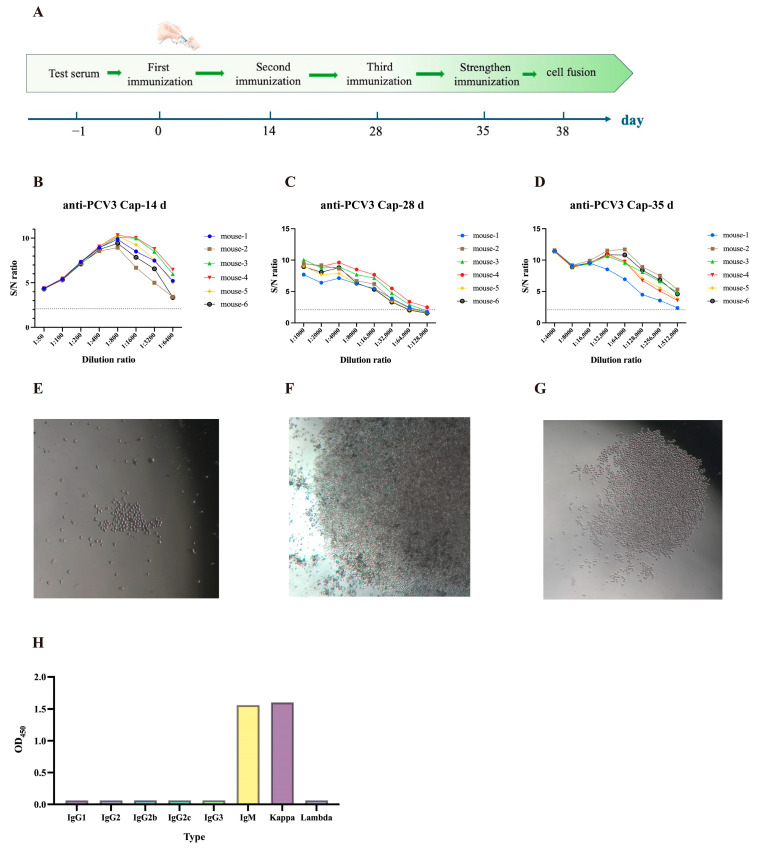
Preparation and Subtype Identification of Monoclonal Antibodies. (**A**) Immunization Schedule. Mice were immunized on days 0, 14, 28, and 35 according to the schedule. Blood was collected via tail vein on the day prior to each immunization, serum was isolated, and the titer of antibodies produced in vivo was determined. (**B**) ELISA results for antibody titers in mouse serum at 14 days post-immunization. (**C**) ELISA results for antibody titers in mouse serum at 28 days post-immunization. (**D**) ELISA results for antibody titers in mouse serum at 35 days post-immunization. S/N ratio = OD_450_ sample/OD_450_ negative sample. The dashed line indicates a ratio of 2.1. Results were considered positive when the ratio exceeded 2.1 (dashed line) and negative when below 2.1. (**E**) 3 days after cell fusion. (**F**) 14 days after cell fusion. (**G**) 7 days after subcloning. (**H**) Identification results for the 3E6 subclass and type of monoclonal antibody. OD_450_ values were measured using a microplate reader. Hybridoma cells were cultured in HAT medium for the first ten days. Expansion and subcloning were performed in HT medium, both at 37 °C in a cell culture incubator.

Using pre-immunization mouse serum as the negative control, mice were immunized with emulsified PCV3 Cap recombinant protein according to a four-dose immunization schedule (Figure 6A). Indirect ELISA results showed that serum antibody titers in immunized six mice exceeded 1:6400 (S/N > 2.1) at 14 days post-immunization, reached approximately 1:128,000 at 28 days, and exceeded 1:512,000 (S/N > 2.1) at 35 days (Figure 6B–D). These results demonstrate that the PCV3 Cap recombinant protein elicits high-titer and stable antibody production in mice, exhibiting excellent immunogenicity.

Following culture of the fused hybridoma cells (Figure 6E,F), three rounds of subcloning via limited dilution (Figure 6G) yielded a stable hybridoma cell line that secretes monoclonal antibodies, which was designated 3E6. This monoclonal antibody has an IgM heavy chain and a kappa light chain (Figure 6H).

### 3.8. Specificity Characterization of Monoclonal Antibodies

**Figure 7:**
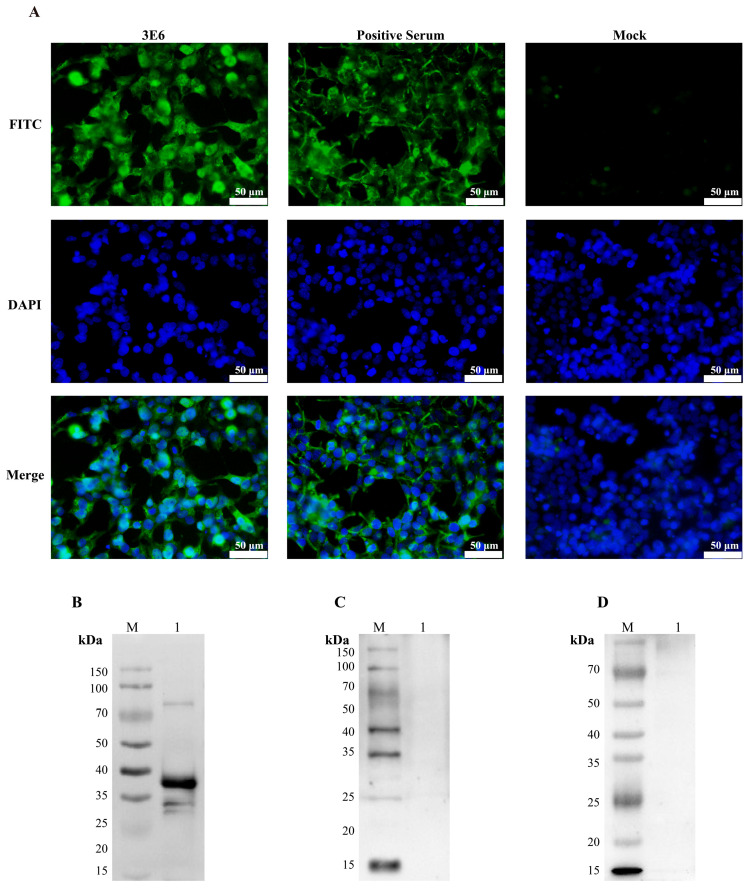
Specificity Characterization of Monoclonal Antibody 3E6. (**A**) Immunofluorescence assay. pcDNA3.1-PCV3 Cap plasmid was transfected into 293T cells, while the Mock group received the empty pcDNA3.1 vector. The primary antibody for both the 3E6 and Mock groups was monoclonal antibody 3E6; the primary antibody for the positive control group was positive serum diluted 1:200 from immunized mice. (**B**) Specific identification of monoclonal antibody 3E6 and PCV3 Cap protein. M: protein molecular weight marker; line 1: PCV3 Cap. (**C**) Specific identification of 3E6 and PCV2 Cap protein. M: protein molecular weight marker; line 1: PCV2 Cap. (**D**) Specific identification of 3E6 and PRRSV N protein. M: protein molecular weight marker; line 1: PRRSV N.

Immunofluorescence results indicate that monoclonal antibody 3E6 specifically binds to 293T cells transfected with pcDNA3.1-PCV3 Cap. FITC fluorescence signals were predominantly localized in the cytoplasmic region. Merge images showed no significant overlap with DAPI-stained nuclear blue fluorescence. The positive Serum group exhibited specific fluorescence, whereas the pcDNA3.1 vector Mock group showed no specific fluorescence (Figure 7A). Further confirming that PCV3 Cap protein, as the viral Cap protein, is primarily expressed and assembled in the cytoplasm. Western blot analysis showed that mouse ascites (1:1000 dilution) specifically bound to PCV3 Cap protein but did not react with unrelated proteins, demonstrating good specificity (Figure 7B–D).

## 4. Discussion

Based on genomic differences, PCV has been classified into four species: PCV1, PCV2, PCV3, and PCV4 [2]. Notably, PCV2 is the etiological agent of PMWS and PDNS, while PCV3 has been associated with PDNS and reproductive failure in pigs [9,14]. Coinfection of PCV4 with other PCV genotypes (PCV2, PCV3, or both) is common in pig herds, and such coinfections have been documented in pigs presenting clinical signs of PMWS, PDNS, and reproductive failure [25,26,27]. Currently, PCV3 and PCV2 pose a more significant threat to the global swine industry. Vaccination is an effective strategy for preventing and controlling PCV infection; however, PCV3 is a recently identified virus, and its pathogenic role remains under investigation.

In clinical settings, PCV3 frequently co-infects with PCV2 and is associated with porcine circovirus-associated diseases (PCVAD) [28]. In recent years, PCV3 research surveys conducted across 17 provinces in China have revealed a PCV3 positivity rate of 31.07%. Jiangsu, Guangxi, and Guizhou recorded the highest positivity rates, all exceeding 66% [29]. Previous studies reported PCV2/PCV3 coinfection rates ranging from 27.6% to 39.39% [21,30,31], and with rates as high as 69.74% observed in a pig farm in Henan Province, China [32]. Additionally, studies have detected PCV4 genomic DNA in pigs across several provinces in China and in South Korea, with positive rates ranging from 1.6% to 45.39% [25,33]. In the present study, qPCR analysis of 1224 clinical samples revealed a higher PCV3 positivity rate (21.90%) than PCV2 (14.13%) in Liaoning Province, with a coinfection rate of 4.08%. Research indicates that seasonal factors influence the infection rates of PCV2 and PCV3 [34]. In addition, PCV3 and PCV2 exhibited distinct seasonal prevalence patterns in Liaoning Province. The prevalence of both viruses and their coinfection peaked in winter, while PCV3 also reached a high positivity rate in summer (notably in July), and PCV2 maintained elevated levels in January. The seasonal prevalence of PCV2 and PCV3 in Liaoning Province is primarily attributed to the combined effects of environmental conditions, porcine immune status, and farm management practices: cold stress in winter impairs pig immunity, and poor ventilation plus high stocking density in enclosed barns facilitate viral transmission, leading to the peak of both viruses and their coinfection. Moreover, in Liaoning, the PCV3 positivity rate is higher than that of PCV2. This regional discrepancy may reflect differences in farming practices and biosecurity measures. PCV2 vaccination has been widely implemented in large-scale pig farms in Liaoning, effectively reducing PCV2 prevalence, whereas the lack of a commercial PCV3 vaccine may have contributed to increasing PCV3 circulation. Phylogenetic analysis demonstrated that all six PCV3 sequences belonged to the PCV3b subtype, consistent with previous reports identifying PCV3b as the dominant genotype in Northeast China [35]. In other Asian countries, early studies reported that PCV3a was the predominant strain circulating in India [36]. High nucleotide and amino acid homology with the Korean strain PCV3/KU-1601 suggests that PCV3 circulating in Liaoning Province may have evolved locally from China’s neighboring countries rather than being directly derived from the original U.S. strain. Beyond breeding practices and vaccine factors, inadequate biosecurity measures at local farms and the heavy reliance on externally sourced breeding stock may also have contributed to the introduction and sustained transmission of PCV 3b.

The Cap protein is the sole structural protein of PCV and contains the major antigenic determinants of the virus. It induces the production of virus-specific neutralizing antibodies and is therefore considered an ideal antigen for the development of genetically engineered vaccines [14,15]. In recombinant vector vaccines, the PCV2 Cap protein has also demonstrated significant application value [17]. In addition, the Cap protein exhibits genetic polymorphism, which plays a critical role in viral attachment, assembly kinetics, and Cap stability [14]. PCV3 has been classified into three genotypes—PCV3a, PCV3b, and PCV3c—with PCV3a further subdivided into PCV3a-1, PCV3a-2, and PCV3a-IM [37]. Previous studies have identified three conserved linear B-cell epitopes in PCV3, located at amino acids 57–61, aa 140–146, and aa 161–166 [38]. Compared with reference strains, the six PCV3 sequences identified in this study harbored eight amino acid substitutions, seven of which were located within predicted B-cell epitopes, including the key sites for subtype classification, A24V and R27K, as studies have confirmed that mutations at these two sites are associated with viral immune escape mechanisms [16]. In addition, PCV3-CN/Liaoning-1 and PCV3-CN/Liaoning-5 exhibited unique amino acid substitutions. These region-specific mutations may be associated with receptor binding or antigenic variation. However, their effects on PCV3 virulence and immunogenicity require further investigation.

Truncated fragments may lack certain conformational epitopes or functional domains present in the full-length protein. However, nuclear localization signals (NLS) are short peptide sequences enriched in basic amino acids [39]. Analysis of the nuclear localization characteristics of the PCV3 Cap protein identified an additional putative NLS in the central region (aa 131–143), in addition to the well-characterized primary N-terminal NLS [40]. The high arginine content of NLS regions increases protein hydrophobicity, thereby impeding the expression of the full-length Cap protein in prokaryotic systems, whereas truncated variants can be expressed with higher efficiency [41]. Consequently, Cap protein variants with truncated NLS regions are commonly used for recombinant expression in *E. coli* and subsequent ELISA-based detection [42,43]. Moreover, previous studies have demonstrated that the PCV3 Cap protein exerts cytotoxic effects on porcine cells, which are closely associated with its nuclear localization [40]. In this study, truncation of the arginine-rich N-terminal nuclear localization signal region of Cap successfully enabled recombinant protein expression in an inclusion body form. This aligns with other findings that truncating the NLS region enhances PCV2 Cap protein expression levels [44]. Immunization of mice with the recombinant protein demonstrated favorable immunogenicity and biosafety after refolding, indicating its potential as a candidate antigen for subsequent PCV3 vaccine development.

The demand for rapid and accurate diagnostic methods in disease surveillance has increased substantially, and monoclonal antibody technology offers distinct advantages in this context. Immunoglobulin M (IgM) antibodies represent a conserved and essential component of the adaptive immune response. IgM molecules polymerize predominantly into pentameric and hexameric structures, enabling high-avidity binding to antigens [45]. During primary immune responses, rapid production of large quantities of antibodies is required for effective neutralization, with IgM predominating at this stage [46]. Compared to IgG, IgM is larger and more complex in structure, and its purification is therefore more challenging [45]. The generation of IgM monoclonal antibodies may therefore reflect the early-stage immune responses elicited by repeated booster immunizations. Although their neutralizing activity requires further validation, IgM monoclonal antibodies meet the key requirements for fundamental research and diagnostic applications. Previous studies have demonstrated that administration of monoclonal antibodies significantly alleviates pulmonary lesions and reduces viral loads in porcine alveolar macrophages of PRRSV-infected pigs, providing valuable insights for immunoprophylaxis [47]. The monoclonal antibody 3E6 produced in this study is an IgM with a κ light chain and specifically recognizes the PCV3 Cap protein without cross-reactivity. Compared with polyclonal antibodies, this monoclonal antibody demonstrates higher specificity and batch-to-batch consistency, supporting its application in Western blot, immunofluorescence assays, and the clinical diagnosis and epidemiological surveillance of PCV3.

## 5. Conclusions

This study investigated the infection status of PCV2 and PCV3 in the Liaoning region and analyzed the genetic evolution of the Cap protein of PCV3. In addition, an ELISA assay for the Cap protein was established, and monoclonal antibodies were prepared. The main limitations of this study are the limited number of Cap gene sequences obtained and the small quantity of monoclonal antibodies produced. Future research will focus on the development of PCV3 vaccines.

## Figures and Tables

**Table 1:** Primer information for PCV3.

Primer Name	Primer Sequence (5′-3′)	PCR Fragment Length (bp)
PCV3-F1	CCACAGAAGGCGCTATGTC	330
PCV3-R1	CCGCATAAGGGTCGTCTTG	
PCV3-F2	CCCACATGCGAGGGCGTTTACC	870
PCV3-R2	CGAGGCCGCTTCATCATCCACT	
Cap-BamHI-F	CGCGGATCCATGAGACACAGAGCTATATTCA	549
Cap-XhoI-R	CCGCTCGAGGAGAACGGACTTGTAACG	

**Table 2:** PCV infection rates in Liaoning Province during 2024.

Month	PCV3 Positive/Total (%)	PCV2 Positive/Total (%)	Coinfection Positive/Total (%)
1	37/118 (31.36%)	29/118 (24.58%)	14/118 (11.86%)
2	44/141 (31.21%)	31/141 (21.99%)	10/141 (7.09%)
3	12/72 (16.67%)	10/72 (13.89%)	4/72 (5.56%)
4	16/85 (18.82%)	12/85 (14.12%)	3/85 (3.53%)
5	14/95 (14.74%)	8/95 (8.42%)	1/95 (1.05%)
6	18/110 (16.36%)	4/110 (3.64%)	2/110 (1.82%)
7	28/80 (35.00%)	10/80 (12.50%)	0/80 (0%)
8	21/74 (28.38%)	5/74 (6.76%)	2/74 (2.70%)
9	8/120 (6.67%)	12/120 (10.00%)	3/120 (2.50%)
10	13/119 (10.92%)	10/119 (8.40%)	0/119 (0%)
11	19/97 (19.59%)	18/97 (18.56%)	4/97 (4.12%)
12	38/113 (33.63%)	24/113 (21.24%)	7/113 (6.19%)
Total	268/1224 (21.90%)	173/1224 (14.13%)	50/1224 (4.08%)

**Table 3:** PCV infection rates across nine cities in Liaoning Province.

Cities	PCV3 Positive/Total (%)	PCV2 Positive/Total (%)	Coinfection Positive/Total (%)
Shenyang	65/215 (30.23%)	54/215 (25.12%)	12/215 (5.58%)
Dalian	52/171 (30.41%)	38/171 (22.22%)	8/171 (4.68%)
Fushun	29/116 (25%)	12/116 (10.34%)	4/116 (3.45%)
Chaoyang	13/122 (10.66%)	8/122 (6.56%)	6/122 (4.92%)
Liaoyang	33/124 (26.61%)	18/124 (14.52%)	3/124 (2.42%)
Fuxin	11/152 (7.24%)	4/152 (2.63%)	2/152 (1.32%)
Jinzhou	35/145 (24.14%)	28/145 (19.31%)	10/145 (6.90%)
Panjin	27/104 (25.96%)	6/104 (5.77%)	3/104 (2.88%)
Anshan	3/75 (4%)	5/75 (6.67%)	2/75 (2.67%)
Total	268/1224 (21.90%)	173/1224 (14.13%)	50/1224 (4.08%)

**Table 4:** PCV infection rates in different organs and tissues in Liaoning Province.

Sample	PCV3 Positive/Total (%)	PCV2 Positive/Total (%)	Coinfection Positive/Total (%)
Lung	28/252 (11.11%)	18/252 (7.14%)	4/252 (1.59%)
Spleen	16/186 (8.60%)	5/186 (2.68%)	2/186 (1.08%)
Kidney	1/5 (20%)	2/5 (40%)	0/5 (0%)
Lymph node	19/74 (25.68%)	8/74 (10.81%)	4/74 (5.41%)
Nasal swab	8/69 (11.59%)	13/69 (18.84%)	3/69 (4.35%)
Serum	196/638 (30.72%)	127/638 (19.91%)	37/638 (5.80%)
Total	268/1224 (21.90%)	173/1224 (14.13%)	50/1224 (4.08%)

**Table 5:** Detection results for ELISA specificity.

Serum	PRV	PEDV	PCV2	PRRSV	CSFV	SVA	PCV3
S/P	0.104	0.088	0.106	0.069	0.051	0.068	0.767
Result	-	-	-	-	-	-	+

PRV, Pseudorabies virus; PRRSV, Porcine reproductive and respiratory syndrome virus; PEDV, Porcine epidemic diarrhea virus; CSFV, Classical swine fever virus; SVA, Senecavirus A.

**Table 6:** Detection results of intra-batch repeatability.

Serum Number	Intra-Batch Replicate Experiments					CV%
	Repeat 1	Repeat 2	Repeat 3	Mean	SD	
1	0.250	0.235	0.242	0.242	0.007	3.01%
2	0.235	0.243	0.234	0.237	0.005	2.09%
3	0.232	0.249	0.237	0.240	0.009	3.63%
4	1.740	1.680	1.681	1.701	0.035	2.03%
5	1.642	1.681	1.532	1.691	0.077	4.77%
6	1.620	1.683	1.525	1.609	0.079	4.94%

**Table 7:** Detection results of inter-batch repeatability.

Serum Number	Inter-Batch Replicate Experiments					CV%
	Repeat 1	Repeat 2	Repeat 3	Mean	SD	
1	0.257	0.238	0.244	0.246	0.010	3.96%
2	0.236	0.237	0.257	0.243	0.012	4.76%
3	0.255	0.238	0.225	0.240	0.015	6.19%
4	1.768	1.730	1.495	1.664	0.148	8.89%
5	1.732	1.611	1.667	1.670	0.061	3.63%
6	1.737	1.664	1.588	1.663	0.075	4.50%

## Data Availability

The data presented in this study are available on request from the corresponding author due to regulations and agreements governing uncharacterized animal sample resources. This study investigated various information and the infection rates of porcine circovirus across pig farms of different scales. While specific farm details and infection data are confidential, the article presents overall infection trends and regional distribution patterns—results that can be publicly disclosed.
